# Factors associated with reporting nursing errors in Iran: a qualitative study

**DOI:** 10.1186/1472-6955-11-20

**Published:** 2012-10-18

**Authors:** Fatemeh Hashemi, Alireza Nikbakht Nasrabadi, Fariba Asghari

**Affiliations:** 1 Fatemeh (P.B.U.H) College of Nursing and Midwifery, Shiraz University of Medical Sciences, Shiraz, Iran; 2 School of Nursing and Midwifery, Tehran University of Medical Sciences, Tehran, Iran; 3 Medical Ethics and History of Medicine Research Center, Tehran University of Medical Sciences, Tehran, Iran

**Keywords:** Nursing errors, Barriers, Motivators, Patient safety

## Abstract

**Background:**

Reporting the professional errors for improving patient safety is considered essential not only in hospitals, but also in ambulatory care centers. Unfortunately, a great number of nurses, similar to most clinicians, do not report their errors. Therefore, the present study aimed to clarify the factors associated with reporting the nursing errors through the experiences of clinical nurses and nursing managers.

**Methods:**

A total of 115 nurses working in the hospitals and specialized clinics affiliated to Tehran and Shiraz Universities of Medical Sciences, Iran participated in this qualitative study. The study data were collected through a semi-structured group discussion conducted in 17 sessions and analyzed by inductive content analysis approach.

**Results:**

The main categories emerged in this study were: a) general approaches of the nurses towards errors, b) barriers in reporting the nursing errors, and c) motivators in error reporting.

**Conclusion:**

Error reporting provides extremely valuable information for preventing future errors and improving the patient safety. Overall, regarding motivators and barriers in reporting the nursing errors, it is necessary to enact regulations in which the ways of reporting the error and its constituent elements, such as the notion of the error, are clearly identified.

## Background

In spite of considerable advances in technology and health care skills, many patients are harmed or die due to the medical errors. In addition, a large number of such errors lead to exorbitant expenditures which have been reported in numerous studies in different countries [1,2]. A considerable portion of medical errors are nurses’ errors while providing health care services, which annually cause thousands of deaths, harm to people, and,as a result, an increase in treatment expenses [3].

In fact, error is a serious, inevitable, and permanent threat to the patient safety [4]. Healthcare providers, despite the precision and mastery skills, are not immune to such errors [5]. For example, in a research conducted at the University of Pennsylvania, 30 percent of the nurses had committed at least one error during the 28 days of the study [6]. In Iran, the incidence of these errors does not seem to be lower than the errors practiced in the health systems of the developed countries.

Evidence for this conjecture comes from the increase in the cases of complaint from physicians and nurses who referred to the Iranian Medical Council and courts [7].

Regardless of making or not making harms to the patients, professional errors represent the system’s vulnerability. One of these problems is the lack of safety culture and undesirable working conditions for both nurses and physicians. In general, system problems can be prevented through reporting 3 types of errors: errors which harm the patients, errors which occur but do not result in patient harm, and errors which could have caused harm but were mitigated just before reaching the patient. Through reporting, different types of errors can be shared and tracked by healthcare providers and ways of reducing and preventing the incidences and reverse events can be taught, as well [8,9]. Therefore, reporting the professional errors for improving the patient safety is essential not only in hospitals, but also in outpatient care centers [8,10,11]. Unfortunately, nursing staff, like many other medical staff, are often reluctant to report their professional errors.

Jolaee et al. believe that medication errors are usually under reported and emphasize studying the importance of barriers in reporting the errors [7]. Therefore, the present study aims to explore the factors associated with reporting the nursing errors.

## Methods

This qualitative study was conducted in the hospitals affiliated to Shiraz and Tehran Universities of Medical Sciences and aimed to clarify the factors associated with nursing errors based on the nurses’ experiences. In general, qualitative methods provide the opportunity for different aspects of a reality to emerge [12].

In this study, the focus group methodology was used in order to investigate the nurses’ thoughts and feelings about the factors associated with reporting the nursing errors. The goal of the focus group methodology is to facilitate discussions about a particular phenomenon among the research participants in a systematic and verifiable manner [11]. This method is the best for data collection, providing rich insights into potentially vulnerable issues, and explaining the beliefs which impose a special behavior [11,13,14]. The inductive nature of the focus group methodology and the quality of the data allowed for the nurses’ experiences to be discerned, organized, and categorized [14].

In the current study, 17 homogeneous focused group meetings were held with the presence of matrons, supervisors, head nurses, and expert nurses in the hospitals of the two universities. Each group consisted of 5 to 7 participants and each session lasted for 60 to 90 minutes [15,16]. After coordination with the Office of Nursing at the Universities, all matrons and supervisors as well as a number of expert head nurses and nurses from different hospital wards were invited to participate in the meetings until data saturation. To guide the focus group discussions, a scenario was developed prior to initiating the study. The questions targeted the nurses’ experiences in confronting the nursing errors and their rationale for and concerns about reporting the errors. This scenario of a nursing error was prepared from literature review [13] in order to be brought forth for discussion in the focus group meetings. The scenario was reviewed and perused by the research team and based on the feedbacks from a few expert nurses, changes were made in order to make it more comprehensible [11]. It should be noted that all the focus group meetings were directed by 2 out of the 3 researchers of the study.

The study team included 1 investigator unaffiliated to the study hospital who led all the focus groups, 1 investigator employed at the study hospital who observed and recorded the field notes during each focus group session, and 1 nursing faculty member who served as experts and mentors during the study.

Prior to the meetings, written informed consents for recording the discussions were obtained from all the participants. The participants were then asked “to whom the occurred error in the scenario should be reported”. Afterwards, the participants made comments about reporting or not reporting the scenario error or the similar ones. In other words, the participants were questioned about their reactions and ways of reporting the error in similar situations and also reasons for reporting or not reporting the error as well as the other similar errors. Then, the researchers used probes as well as clarifying questions in order to open the subject on a broad level and provided the participants with the opportunity to put their actual behavior and experiences regarding the scenario issues into dialogue in a free and creative atmosphere. An audio recording was also made from each session's discussions.

Inductive content analysis was used in order to determine the main themes, categories and sub-categories of the factors associated with reporting the nursing errors.

The data were evaluated word-by-word by 2 of the study investigators and then discussed among all the team members in order to identify the themes representing the nurses’ perceptions and experiences about reporting the nursing errors. The data quality and procedural effectiveness were monitored by a team member who did not participate in the focus groups. In this step, the categories which embraced the experiences were developed [17,18]. The first and second authors were involved in the process of developing the categories, whereas the third author confirmed the developed categories. It should be noted that the first and second authors were experienced in the field of nursing errors and qualitative methodology.

The analysis was carried out in several steps, the first of which included several readings of the text as a whole in order to arrive at the overall understanding of the text. The first, second, and the last author independently carried out the overall readings and then met and discussed their impressions of the text. In the next step, meaningful units related to the aim of the study were identified and marked. Finally, the meaningful units were separately condensed by the first and the second author and were then discussed for final agreement [12,17,18].

In case of disagreement between the researchers, the transcription were read and analyzed again and the final agreement was reached in a separate meeting with the presence of all the researchers. After all, the derived themes are supported with the participants' quotations.

### Ethical considerations

Describing the purpose of the research, obtaining written informed consent forms for taking part in group discussions and recording their voice from all the participants, commitment to share the findings if desired, maintaining the anonymity, and the participants’ being able to withdraw at any stage of the research were the ethical considerations of this study. This research is conformed to the Helsinki Declaration http://www.wma.net/en/30publications/10policies/b3/) and was approved by the ethical committees of Tehran and Shiraz Universities of Medical Sciences.

## Results

The study participants included 115 nurses with working experience of 3 to 27 years and age range of 23 to 54 years. Eighty nurses and head nurses were working in general and specialized internal and surgical, intensive care, pediatrics, neonatal, psychiatry, emergency and trauma, and obstetrics and gynecology wards as well as the specialized clinics and 35 matrons and supervisors were working in all hospitals and specialized clinics of the two universities. Most of the participants (n = 111) had bachelor's degrees in nursing and the rest had M.Sc. degrees.

After data analysis, themes or main categories of the factors related to reporting the nursing errors were a) the general approaches of the nurses towards practice errors, b) barriers in reporting the nursing errors, and c) motivators in reporting the errors. Besides, each main class contained other sub-classes which explain as follows:

### The general approaches of nurses towards the professional errors

The participants had perceived individual (person), system, or a combination of person and system approach which affected their reactions and functions regarding the error. The participants with person approach thought that nurses should not have any slips or lapses. They also believed that if an error is committed by an individual, she or he is responsible and must be punished. For example, an expert nurse said: "*As far as I am concerned, the nurses’ errors are unacceptable. In Our job, there is no way of making mistakes. We are dealing with the people’s lives; so, no mistake is accepted*”. Another participant said: "*Nurses who commit an error must fill out the special accident form and must be punished because of that error*”. The participants with system approach believed that nurses, like others, are susceptible to error. They also considered error as a multifactorial event and believed that in many cases, an error is the product or consequence of the flaws and shortcomings in the organization. This group was reluctant to put an individual at the sharp end for doing the wrong and did not believe in counting him guilty. This statement from a young nurse represents the viewpoint of a great number of the participants of this group: “*Now, people think that we, as nurses, should not make mistakes, while anyone might make mistakes. There are errors in all lines of work. Moreover, when a person makes a mistake, it’s not only because of him, it can have many reasons*”.

Some participants’ approach was a combination of the person and system approaches. This group thought that although many flaws and shortcomings in the organization have a role in error commission by an individual, features, such as knowledge, skill, responsibility, and accountability of the nurses, are important in reducing the likelihood of error. These participants believed that problems and deficiencies in the organization do not acquit the nurses of an occurred error and the problems which threat the patient safety. One participant said: "*Despite all the problems, I still believe that every individual should be responsible and accountable for his/her mistakes*”. This study showed that older participants with more years of working experience, matrons, and supervisors had a person approach, while younger participants with a few years of working experience, nurses, and head nurses had a system or a combination of person and system approaches towards the nursing errors.

### Barriers in reporting the errors

In this study, the nurses complained about some factors as inhibitors in reporting the errors, which were placed in the barriers class. For example, the factors associated with nurses, organization, and the nurses’ perception of the incidence and consequences of the error were stated as the main barriers in reporting the errors.

a) Factors associated with nurses: are the participants’ perceptions which stop them from reporting the errors to the authorities as well as the in-charge physicians. These factors include fear of legal action and job threats, fear of economic losses, fear of honor and dignity, weakness of knowledge, weakness of nursing skills in error management, and unwillingness to accept the responsibility of the errors. A selection of the study participants’ statements is as follows: “*If we report errors, they will be used against us. There will be no legal protection. They threaten us. Our problems will be examined in the Medical Council. Physicians will vote against us and in favor of their own interest. We are afraid of being ousted from our job. Continuous warning comes from the nursing office*”. “*A nurse had to pay $8000 as blood money because of a mistake which was not only his fault. How much is our income to pay blood money*”. “*We are not treated right, we are humiliated. They establish feelings of incompetence in us*”. “*Someone may not be aware of his mistake and does not report it*”. “*Nurses have little knowledge in this field. The educational system has shortcomings*”. “*The personnel are different; it depends on an individual’s sense of responsibility and commitment to work*”.

b) Nurses’ perception of the incidence and consequences of the errors: The factors classified in this group were based on the participants’ experiences and error perceptions which stopped them from reporting the errors. These factors included the impact degree as well as the severity of the error, not having adverse events, and ambiguity in the notion of the error. These sentences were frequently heard from the participants: “*When an error with no serious outcome is practiced and everything is under control, no report is needed*". Or "*something might be right in my opinion, but you or the head nurse considers it as an error! We should know what an error is!! For example, if a patient is drugged with delay due to the crowdedness of the wards, is it an error*?"

c) Organizational factors: The majority of the participants acknowledged that there are factors in their organization which tempt them to keep errors and mishaps unreported. Unpleasant behaviors and previous inadequate reactions of the organization, including the managers, physicians, and colleagues, and also the inappropriate reaction of the manager regarding the impact and intensity of the error lead to under-reporting the errors and covering them up. What follows is a selection of the statements provided by the study participants: “*When we report our errors, we face harsh and unfair behaviors (warnings, threats,* etc.*). Should an error be reported in such a milieu?!*” Another major concept associated with the organization which stopped the nurses from reporting the errors was shortcomings in the safety culture, which appeared in the form of the limited threshold of fault tolerance, nonexistence of team response, assigning one person at the sharp end, and name, blame, and shame culture. In this regard, the authorities', physicians', managers', and other team members' flying into rage and shirking from the error responsibilities as well as the nurses' being responsible, being beaten by the patients’ loved ones, going to court, being at the sharp end, being named, blamed, and shamed, and being humiliated were expressed by the participants.

d) Work pressure/high load of responsibility: The factors classified in this group were based on the participants’ perceptions of high work load and pressure as well as the responsibilities of the nurses which caused the errors to go unreported. These factors include the personnel’s lack of time and the reporting process’ being time-consuming.

A selection of the participants’ statements is: “*We are really busy; a great number of patients and a limited number of staff. We do not have time for reporting the errors and being involved in the process of error reporting. Doing something for the patient in this little time is my concern*”.

### Motivators in error reporting

a) Factors associated with nurses: These factors include the nurses' knowledge and skills in managing the errors, responsibility, professional commitment, and professional accountability. What follows is a selection of the nurses’ experiences in this regard: "*Educated and skilled nurses report their errors*”. "*The educational system should be effective*". "*It depends on an individual, being responsible is important*”.

b) Factors related to errors: Here, a clear definition of error, its consequences and negative adverse events, and the profitability of the reports for the patients were considered as motivators. The following experiences were expressed by the study participants: “*Serious errors should be reported. When my wrong deed caused injury to a patient and it is obvious, it must be reported*”. "*What to be reported should be clearly defined*”. Or “*If reporting is effective in the patients' recovery, I’ll report the error*".

c) Organizational factors: Based on the participants' experiences, the factors of this class were related to their work place and facilitated reporting the errors. Dominant supportive atmosphere, no authority and physician shirking from the responsibility of the practiced error, and anonymous reporting system were the issues raised by the participants. The nurses said: “*In our hospital, the physicians don’t leave us alone in case of an error; we solve it together*”. Or "*If a person’s name is not important in a report and we can report anonymously, it will be perfect*”. Or "*In case an error occurs, we need them to understand us; so that we dare to report the errors*". Or "*In case of errors, we have to search for the reason rather than the guilty individual*". Or "*The physician and the head nurse should be accountable, as well*".

In spite of different fears and threats which stopped the participants from reporting the errors, they believed that data exchange on errors and reporting the errors are among the duties of the nursing staff and can provide effective post-error care and treatment and prevent further harms or injuries. They also believed that reporting the professional errors are learning treasures which can work in favor of other nursing staff and reduce the likelihood of error.

## Discussion

In the present study on reporting the nursing errors, three main themes with several sub-classes were defined. One of the extracted themes was the participants’ general approach towards the error. Older nurses and nursing managers had a person approach towards the nursing errors, while younger participants had a system approach or a combination of these two approaches. As Reason pointed out, human errors can be viewed by both person and system approaches each of which has different philosophies and reasons. The person approach assumption is that wrong things are done by wrong people. It seems that unsafe actions arise from some of the individuals' mental processes, such as forgetfulness, lack of motivation, carelessness, and negligence. In the system approach, humans are fallible and errors are to be expected even in the best organizations. Besides, in case of an error, how and why are important rather than who [19]. However, each person and system approach has different philosophies and reasons regarding the human errors and understanding these differences is important in dealing with them. Perhaps a combination of the two approaches improves reporting and ideally results in safer processes. This means that always a set of conditions provides the possibility of an error, but identifying the causes of the error is necessary for averting similar errors from harming future patients. Of course, it should be noted that changing the human conditions is impossible, but changing the working conditions is practical.

The next major theme in this study was the barriers in reporting the nursing errors. In fact, organizational improving programs could face obstacles. Reporting the nursing errors is an essential activity for improving patient safety, but always some factors lead to its reduction [8,11,20]. Based on the participants' perceptions, barriers in reporting the professional errors in nursing were classified into 4 sub-classes of nurse-related, error-related, organization-related, and high work load factors. Although the present study was conducted on reporting the nursing errors, the three identified sub-classes which had been considered as barriers by the participants were the same as the 3 factors on error disclosure; i.e., nurse-related, error-related, and organization-related errors, which were found in the study of Fein et al. [13]. This might be due to the similarity of the nature and reasons of reporting the professional errors to authorities to those of error disclosure to patients. In other words, disclosing errors to the patients and their loved ones is actually another way of reporting the errors [8].

Different fears, such as fear of legal action and job threats, fear of economic losses, fear of honor and dignity, and fear of reporting outcomes [8,21-23], and also some personal characteristics of nurses, such as lack of professional responsibility and accountability and lack of knowledge and skill in how to report errors, are the most prominent barriers in reporting the errors in the sub-class of factors related to nurses. Therefore, conscience, commitment, sense of personal responsibility, and the authorities' not considering the nursing errors as crimes, supporting the error reporters, and not reinforcing fear in those who have committed an error will increase reporting the errors. Training and encouraging the nurses to identify and report the working errors in a non-punitive milieu will increase error reporting, as well [8,13].

Factors associated with errors are among the other identified sub-classes in the main theme of barriers in reporting the errors. Although the participants of the present study did not have a universal agreement on the effect of these factors, the majority considered the impacts and consequences of errors as important issues in the nurses' decision to report or not, which is consistent with the results of a large number of other studies conducted on this issue. Based on the participants' perceptions in this study, errors or near misses which did not harm the patients [21,24-28] as well as the ambiguity in the notion of error [29] stopped them from reporting. Moreover, reporting the errors of no adverse outcomes depended on no harsh, threatening, and aggressive confrontation of the authorities. Overall, reluctance of the healthcare team members towards reporting the errors of no adverse outcomes may be due to the various fears and threats felt by the ones who commit the errors [22,30,31] or lack of support from colleagues and authorities for the ones who report the errors [4]. However, reporting near misses is highly important in preventing and reducing the likelihood of error in future and increasing the patient safety [7,8].

Factors related to organizations in the present study are among the other sub-classes of obstacles in reporting the errors. Intolerance toward the errors [13], inadequate reaction of the authorities and colleagues towards the error as well as the individual who practiced that error [16,23,31,32], inappropriate reaction of the authorities regarding the severity of the error [29,31], and a culture of blame and shame [30] are among the threatening factors and barriers in reporting the errors. Unfortunately, putting an individual at the sharp end of an event and individual blaming culture are the most prominent reactions to the error and the one who practiced the error among the providers and, particularly, nurses [8]. Based on the perceptions of the participants of this study, in case an error occurs, even when the nurses are not guilty, the blame is shifted to them. Such unjust actions can cause psychological trauma to the nurses and create fears in the nursing community. Also, damages from the past mistakes which remain in the minds of healthcare providers can limit the tendency toward error reporting and disclosing [8].

The participants of the present study related error reporting to the previous feedbacks from the authorities and colleagues. This means that if the reactions and responses were not in accordance with the error commission or if they had not been supported, they would prefer to under-report the error and cover it up. Overall, lack of support from health care authorities parallel to criminalization of healthcare mistakes [8] increase various fears in the providers [8,33]. Therefore, it is necessary that the remaining damages and fears in the minds of health providers be replaced with efforts to encourage error reporting in a safe and non-punitive environment [4,8].

In this study, high work load and responsibilities are among the other sub-classes of the central theme of barriers in reporting the nursing errors. In the study conducted by Elder et al., also, high work load and responsibilities were considered as the most common barriers in error reporting [11]. These were considered as barriers to disclose and report errors in other studies, as well [13,34]. The participants of this study recommended the involvement of the authorities and physicians in the process of response (team response), while the participants of others researches recommended shifting the responsibility of error reporting to the organization's specific reporter [11].

The next major theme in this study was the motivators in reporting the nursing errors. Motivators in reporting errors encourage the nurses to report the nursing errors. Similar to other studies conducted on the issue, nurses' knowledge and skills to deal with the errors [13], the nurses' personal characteristics, such as responsibility, clarity in the notion of the errors which should be reported [11], and negative impacts and consequences of the errors [13] were among the motivators mentioned in the present study [11,13]. Based on their perceptions, these were classified in the sub-classes of the factors related to nurses and errors.

In other studies, error reporting profitability in various fields, such as adjusting its consequences, improving patient safety, and learning opportunities, was considered as the motivator [4,8,11,21]. Nevertheless, the participants of this study only considered the profitability of error reporting for patient safety and patient recovery as the motivators in reporting the errors. The difference in the attitudes of the participants of the present study to those of the other studies is perhaps due to the absence or little presence of error reporting issue in our clinical settings. This means that if the nurses are educated regarding the error reporting, its related factors, and its profitability by Continuing Medical Education (CME) Centers, they will consider the benefits of error reporting, such as self -training and others-training, support for reporters, getting regular feedback, and improving the quality of health services, with wider range of information and more open-mindedly [11]. Overall, based on the attitudes and perceptions of the participants of this study, organization safety culture is among the factors influencing the nurses' decisions to report the professional errors. In fact, lack of or defected safety culture hinder error reporting, while existence of safety culture will strengthen the nurses’ motivation to report the errors. The factors reducing the safety culture in this study were authorities' and colleagues' intolerance of error, lack of professional support, blaming and shaming the one who committed the error, putting an individual at the sharp end of an event, lack of support for the error reporters by the authorities, the authorities' blaming and punishing the reporters, and lack of the physicians' cooperation as well as team accountability. Wolf et al. also believe that the clinicians who work in a system of blaming and punishing do not report errors due to the fear of being punished. A long standing tradition in healthcare domain is repeating a common expressions of “your name, you are guilty, you're ashamed”. Fear of retaliation and punishment leads to silence, while silence is destructive. In fact, healthcare professionals need to put the important issues and problems of their working environment, such as errors and unsafe actions of their colleagues, into dialogue [8]. When individuals and organizations are able to shift from blaming and shaming culture to a safety culture where name, blame, and shame approach is removed, disclosing and reporting is encouraged, and damages and fears remained in the minds of health service providers are replaced with encouraging error reporting in a safe and non-punitive environment [4,8,29], organizations will be able to have formal error reporting systems and increase the reporting of all types of errors [4,8].

In addition, error reporting can be increased by the authorities' support of those who report the errors and training the staff about the objectives of error reporting [35]. To increase error reporting, health care providers and others should know that first, reporting without penalty leads to improving safety and second, errors are primarily the product of the organizations' flaws [36]. In addition, perfect communication and collaboration between healthcare providers are requirements of a safe environment and in a safe culture; open communications, error reporting, and team accountability among all healthcare providers are facilitated and will be considered as a rule.

Team accountability does not let the responsibility load and the tensions of reporting fall on an individual's shoulder and this is what the present study, like other studies, identified as the reporting motivators [11]. Moreover, anonymous error registration system, which was suggested by some of the participants of the present study, leads to reduction in the responsibility load and results in error reporting reinforcement [8,11].

## Conclusion

Professional errors are indicative of flawed systems and indicate the lack of safety culture and poor working conditions for nurses. System problems can be prevented through reporting of all types of errors. In addition, primarily consideration of error reporting by systems provides extremely valuable information for prevention of future errors. Overall, considering the obstacles and motivators in reporting the nursing errors, it seems necessary to enact regulations in which the ways of reporting errors and their constituent elements, including the notion of the error, are clearly defined. Furthermore, training the nurses as well as the nursing managers regarding the objectives of error reporting and method of using the information of the occurred errors is suggested in order to improve the patient safety and the quality of care in nursing domains. In this case patient safety and work safety for nurses are improved.

## Competing interests

The authors declare that they have no competing interests.

## Authors’ contributions

FH carried out the conception, design, data collection, analysis, and interpretation of the data and drafted the manuscript. AN and FA participated in data gathering and interpretation of the data and helped to revise the initial draft. All the authors read and approved the final manuscript.

## Authors’ information

Mrs. F H is a faculty member Fatemeh (P.B.U.H) College of Nursing and Midwifery, Shiraz University of Medical Sciences, Shiraz, Iran. Her main interest is nursing ethics and oncology nursing and has published a few articles on these issues. She is the corresponding author of this article and can be reached at: Fatemeh (P.B.U.H) College of Nursing and Midwifery, Shiraz University of Medical Sciences, Shiraz, Iran 71936–13119.

Dr. A N is an associate professor at School of Nursing and Midwifery, Tehran University of Medical Sciences, Tehran, Iran. His main interest is nursing education and nursing ethics. He has also published several articles on these issues in both national and international journals.

Dr. F A is MD and assistant professor at Medical Ethics and History of Medicine Research Center, Tehran University of Medical Sciences, Tehran, Iran. Her main interest is medical ethics and has published articles in this regard.

## Pre-publication history

The pre-publication history for this paper can be accessed here:

http://www.biomedcentral.com/1472-6955/11/20/prepub
